# A systematic review and integrative sequential explanatory narrative synthesis: The psychosocial impact of parenting a child with a lysosomal storage disorder

**DOI:** 10.1002/jimd.12482

**Published:** 2022-02-24

**Authors:** Sadie Hassall, Debbie Michelle Smith, Stewart Rust, Anja Wittkowski

**Affiliations:** ^1^ Division of Psychology and Mental Health, School of Health Sciences, Faculty of Biology, Medicine and Health University of Manchester Manchester UK; ^2^ Greater Manchester Mental Health NHS Foundation Trust Manchester UK; ^3^ Manchester Academic Health Science Centre Manchester UK; ^4^ Royal Manchester Children's Hospital, Paediatric Psychosocial Service Manchester UK

**Keywords:** inherited metabolic disease, orphan disease, paediatric, physical health, rare disorder

## Abstract

Lysosomal storage disorders are rare multiorgan, degenerative conditions requiring invasive treatment. Rare disorders pose unique challenges; therefore, exploring their impact is crucial for understanding family needs. This novel review aimed to understand the psychosocial outcomes for parents of children with lysosomal storage disorders. Five electronic databases were systematically searched. Thirty‐eight (23 qualitative, 10 qualitative and 5 mixed methods) studies were included, analysed using a sequential explanatory narrative synthesis and appraised for their methodological quality. Quantitative data revealed the multifaceted impact on parents' psychological and social wellbeing. Qualitative data informed the challenges that these parents faced which were expressed within three main themes: (a) *Uncertainty and the unknown*, (b) *All‐encompassing impact* and (c) *Finding a way forward*. The synthesis demonstrated that factors associated with the condition (symptoms, behaviour and severity) had a substantial negative impact on parental outcomes, upheld by concurrent loss (deterioration and poor prognosis) and uncertainty. This substantive integrated review revealed considerable unmet parental psychosocial needs.

**Abbreviations**
LSD: lysosomal storage disorder
IMD: inherited metabolic disease
PKU: Phenylketonuria
MPS: mucopolysaccharidosis
MPS I: mucopolysaccharidosis type 1
MPS IH: mucopolysaccharidosis type 1 (Hurler syndrome)
MPS IH/S: mucopolysaccharidosis type 1 (Hurler Scheie)
MPS II: mucopolysaccharidosis type 2 (Hunter syndrome)
MPS III: mucopolysaccharidosis type 3 (Sanfilippo syndrome)
MPS IIIa: mucopolysaccharidosis type 3 (Sanfilippo syndrome type a)
MPS IIIb: mucopolysaccharidosis type 3 (Sanfilippo syndrome Type b)
MPS IV: mucopolysaccharidosis type 4 (Morquio syndrome)
MPS Iva: mucopolysaccharidosis type 4 (Morquio syndrome Type a)
MPS V: mucopolysaccharidosis type 5 (Scheie syndrome)
MPS VI: Maroteaux‐Lamy syndrome

## 1 INTRODUCTION

Lysosomal storage disorders (LSDs) are a rare subgroup of inherited metabolic diseases (IMDs), caused by inborn errors in metabolism.^1^ LSDs are heterogenous neurodegenerative disorders that commonly affect major peripheral organ systems and, in some conditions, the central nervous system.^2^ There are more than 50 types affecting approximately one in 5000 live births worldwide.^3^

Rare diseases, such as LSDs, are complex in nature, often harbouring a significant burden of care needs—medically, socially and psychologically.^4^, ^5^ A scoping review of 29 studies^5^ explored the supportive care needs of parents of children with rare diseases, which included epilepsy and chronic illness, discussing the significant impact of caregiving and parental responsibilities on the emotional and social support needs and experiences of parents.

Few reviews have reported on the impact of IMDs on parents; however, cross‐sectional studies have demonstrated that parents of children with a metabolic disease had a significantly poorer Health Related Quality of Life (HRQOL) than other subgroups, such as diabetes.^6^ According to Amber et al,^7^ the type of IMD is key when examining HRQOL of parents. HRQOL in parents of children with Phenylketonuria (PKU) and galactosemia was comparable to that of parents with healthy children and better than other IMDs.

LSDs are commonly diagnosed clinically following symptom‐onset,^8^ which has been found to increase parental psychological distress compared with diagnoses of IMDs via new‐born screening.^9^ Furthermore, LSDs carry invasive treatment options, including enzyme replacement therapy (ERT) and haematopoietic stem cell transplantation (HSCT^10^). HSCT carries a risk of mortality in up to 16.2% of patients, rising to 42.5% if patients required incubation,^12^ thereby increasing parental stress, anxiety and depression.^11^

The reviews focusing on living with a LSD largely lack reporting of parental data, yet the rare diseases literature suggests that there is likely to be a significant psychosocial impact. The aims of this review were to synthesise the existing literature and to enhance our current understanding of the psychosocial consequences for parents caring for a child with a LSD.

## 2 METHODS

This mixed methods systematic review and sequential explanatory narrative synthesis^15^ was informed by the Preferred Reporting Items for Systematic Reviews and Meta‐Analyses guidelines (PRISMA^16^). The review protocol was registered with PROSPERO (212161).

### 2.1 Search strategy and identification of studies

The PICO framework (Population, Intervention, Comparison and Outcome^17^) was used to develop a systematic search strategy for quantitative, qualitative and mixed methods research (see Table S1). The search was conducted electronically in EMBASE, Medline, PsychInfo, CINAHL and Web of Science in June 2020 and updated in March 2021. No restrictions were placed on date of publication.

**FIGURE 1:**
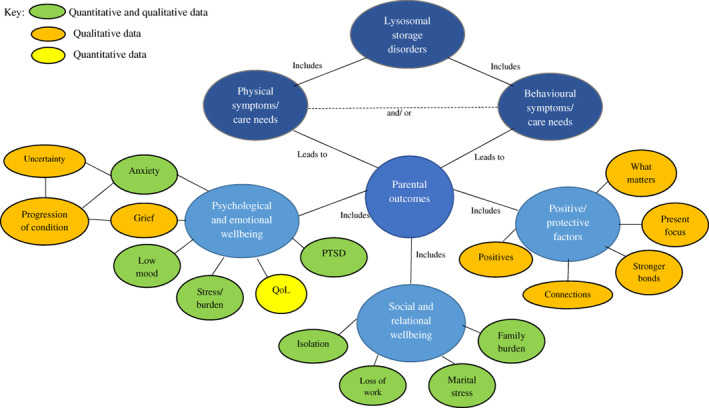
Concept map of results to demonstrate the relationship between findings. PTSD, posttraumatic stress disorder; QoL, quality of life

All references were exported to Mendeley Reference Manager and duplicates were removed. Studies were screened by title and abstract against the inclusion criteria and retained papers were subjected to a full text review. Reference lists and citations of papers, which met the inclusion criteria, and relevant reviews were hand searched for additional studies. Figure 1 outlines this process (see Supporting Information).

The first author undertook the search and 10% of search results (after removal of duplicates) and a further 10% of retained full papers were screened independently by a peer independent of the research team to reduce any potential bias. Any disagreements were discussed and resolved. All authors reviewed the full papers and agreed their inclusion.

### 2.2 Inclusion criteria for studies

Studies were included if they (a) measured at least one psychosocial factor for one or both parents or primary caregiver, (b) were available in English, (c) used quantitative or qualitative methods to collect data, (d) were published in a peer‐reviewed journal, (e) focused on parents of children with a LSD (or data for LSDs were distinguishable from other conditions) and (f) were an original empirical study. Psychosocial impact was defined as psychological or social factors (including social support, employment and relationships) as a direct impact of caring for their child with a LSD.

### 2.3 Data extraction

Data from included studies were extracted and tabulated (see Table S2) by the first author and checked for consistency and accuracy by two experienced reviewers within the research team (AW/DS).

### 2.4 Methodological quality assessment

Studies were assessed for methodological quality and risk of bias using the Quality Assessment Tool for Studies with Diverse Designs (QATSDD^19^), for which good reliability and validity were reported. The tool allowed for 16 ratings on a Likert scale from 0 to 3 (0 = not at all, 1 = very slightly, 2 = moderately and 3 = complete) which allowed for a score by criterion and overall score to be summed; overall scores were converted to a percentage.^19^ Using guidance from previously published reviews,^20^ each study was given a rating of high (>80%), medium (50%‐80%) or low (<50%) to provide an overall indication of methodological quality and associated risk of bias. See Table S3.

All studies were independently rated by the first author and an independent peer. Any discrepancies between ratings were discussed and a final rating was agreed.

### 2.5 Data extraction and synthesis approach

As part of the preliminary synthesis, relevant data were extracted from studies and tabulated according to study methodology (eg, quantitative, mixed or qualitative) and ordered by LSD type. Each study methodology was then synthesised separately in accordance with sequential explanatory designs for mixed methods systematic reviews.^15^ Data from mixed methods studies were analysed in accordance with study methodology (ie, quantitative or qualitative). Quantitative data were analysed first using the grouping of the data to establish themes emerging from patterns. Qualitative data were subsequently analysed to further inform quantitative findings. A thematic analysis^21^ of qualitative data was conducted by two reviewers (SH and DS) independently by extracting relevant data from whole results sections electronically into Microsoft Word. Data were subjected to line‐by‐line coding. Codes were clustered and those conveying the same ideas were combined before themes and sub‐themes were interpreted and defined by the wider research team.

A narrative synthesis approach was applied to the data following adjusted systematic steps outlined by Popay et al^22^: (a) developing a preliminary synthesis, (b) exploring the relationships within and between studies and (c) appraising the robustness of the synthesis. Finally, quantitative and qualitative data were synthesised and a concept map was developed.

## 3 RESULTS

The search identified 2595 references. After duplicates were removed, 2327 were screened, of which 153 publications were retained for a full text review. Thirty‐one studies met the inclusion criteria. Reference lists and citations of these 31 studies were hand‐searched which identified a further seven studies. Thus, 38 papers were included: 23 quantitative, 10 qualitative and 5 mixed methods studies (see Figure 1). A Cohen's Kappa statistic was calculated using SPSS to check inter‐rater reliability of the screening process; inter‐rater reliability was highly acceptable for the initial screen (*k* = 0.855, *P* < .001, rated as ‘perfect’) and the screen of full texts (*k* = 0.646, *P* = .001, rated as ‘substantial’^18^).

### 3.1 Study characteristics

Characteristics of each included study are outlined and summarised in Table S2. Most studies were conducted in Europe (n = 19); seven studies involved cross‐country participants and one study did not specify participants' geographical location. Most studies focused on parents of children with differential MPS diagnoses (n = 29), followed by Batten disease and Pompe syndrome (n = 3, respectively), and Gaucher disease, Alpha‐Mannosidosis, cystinosis and Niemann‐Pick disease (n = 1, respectively).

Sample size was diverse across study designs: quantitative studies ranged from nine to 258 parents/caregivers; qualitative studies ranged from eight to 38 parents and for mixed methods samples ranged from 23 to 93 parents. Studies varied in their reporting of demographic data: parent/caregiver age was most commonly reported (n = 11), followed by education (n = 7), marital status (n = 6), parental ethnicity (n = 6) and employment (n = 2). Twenty‐one studies did not report demographic information for parents/caregivers.

There was variation in how the psychosocial impact of parenting a child with a LSD was explored, largely clustered around the broad concepts of psychological and social outcomes. Quantitative data relied on a variety of different validated (n = 31) and study specific (n = 11) measures. The most frequently used measure was the Social Economic Burden and Health‐Related Quality of Life in Patients with Rare Diseases in Europe (BURQOL‐RD^23^), but it was only used across three studies. In quantitative studies, the most common method of study design for the parent arm of the studies was cross‐sectional (n = 25), followed by cohort studies (n = 2) and one case series. Qualitative data were most commonly collected via interviews (n = 11), followed by surveys (n = 3) and focus groups (n = 1). The most common qualitative methodology were grounded theory and thematic analysis (n = 3, respectively), followed by content analysis (n = 2) and qualitative case study and phenomenology (n = 1, respectively). Five studies did not specify which method was used.

### 3.2 Methodological quality appraisal of studies

Based on overall scores, methodological quality of the included studies varied across study type, yet most studies reported on relevant criteria (see Table S3). Overall, the quality of quantitative studies was rated as ‘medium’ (n = 13) and ‘low’ (n = 10), with no studies being deemed high quality, with a mean quality rating score of 50%. The five included mixed methods studies were rated as ‘medium’ (n = 3) and ‘low’ (n = 2) quality, with a mean score of 59%. In contrast, the quality of qualitative studies was stronger, rated as ‘high’ (n = 1) and ‘medium’ (n = 8), with only one study being rated as ‘low’ (n = 1): mean overall score of 70%.

There was a highly acceptable agreement between the two independent raters on overall methodological quality category, as calculated by Cohen's Kappa (*k* = 0.807, *P* < .001).

### 3.3 Synthesis

#### 3.3.1 Quantitative data

Upon analysing the quantitative data, data were grouped within the themes of (a) psychological and (b) social consequences for parents.

##### Theme 1: Psychological consequences

Psychological impact, measured in 14 quantitative studies and one mixed methods study,^68^ commonly referred to depression, anxiety, stress, posttraumatic stress disorder (PTSD) and quality of life across different LSDs. The outcome measures varied widely across studies: a total of 16 different validated measures were used. Four studies utilised study‐specific and non‐validated measures, three of which were rated as methodologically poor.^39^, ^40^, ^54^, ^67^

Anxiety and depression were assessed in seven studies and were frequently experienced by parents. Higher mean anxiety and depression scores were identified for parents of children with MPS II, MPS III and Batten disease when compared with a comparison or reference group.^28^, ^42^, ^51^, ^57^ In contrast to this, although Grant et al^42^ established that parents of children with MPS III had elevated scores probable of difficulties with anxiety and depression, this was not dissimilar to the comparator group of parents of children with an intellectual disability. This discrepancy could result from the comparator group in Grant et al's sample demonstrating similar difficulties (ie, ‘challenging behaviour’) than comparator and reference groups used in the other studies. Contrastingly, within Alpha‐mannosidosis, mean scores of anxiety and depression were not above clinical threshold. However, conclusions cannot be drawn given the small sample size of only nine parent/caregivers.^65^

Increased parenting stress was identified across different MPS subtypes,^34^, ^40^, ^68^ and was found to be higher than that of parents of children within an oncology service.^2^ Despite this, Lehtonen et al^34^ found that parenting stress in MPS IH was no different to that of the normal population when considering raw scores. Hoffman et al^24^ reported that the incidence of parenting stress was related to different symptoms, with agitation, aggression and sleep disturbance being associated with severe parenting stress. Stress of caregiving was also explored in carers of children with Alpha‐mannosidosis who reported high levels of stress in relation to care provision^65^ on the Carer Stress Index (CSI^66^). PTSD symptoms specifically of parents of children with MPS III were reported in one study,^28^ with 26.9% of mothers and 15% of fathers reporting higher symptoms compared with a reference group.

##### Theme 2: Social consequences

Social factors were measured in relation to caregiving time; impact on work, social life and relational impact in 16 studies (see Table S2). There was a clear impact on parents' working lives, resulting in high levels of unemployment or a reduction in working hours in order to care for their child. Guffon et al^38^ reported that the loss or change to employment frequently affected more mothers compared with fathers.

Time spent caring was considered in six studies and ranged from 9.4 to 4.4 hours per day on average.^25^, ^26^, ^65^ Differences in reporting hours and increased time could be due to the age range used in studies, as parents reported a higher care burden children age and require more support, such as the use of a wheelchair (eg, 40, 63).

Having a child with a LSD also affected the family system. Parents of children with MPS II reported a greater overall family impact, poorer family cohesion and a deterioration in family relationships in comparison to other paediatric groups.^39^, ^46^, ^67^, ^69^ The severity of the condition was found to correlate with this score in both MPS and Batten disease alike; thus, the more severe the condition the greater the family impact and reduced quality of life.^55^, ^57^, ^69^

#### 3.3.2 Qualitative data

Data from nine qualitative studies and qualitative data from five mixed method studies was explored using thematic analysis. Three main themes and ten subthemes were identified.

##### Theme 1: Uncertainty and the unknown

A common theme, which emerged from the data, was the uncertainty that parenting a child with a life‐limiting condition brings. This theme was expressed in various guises, such as the process of diagnosis, treatment and the progression of the disease.

###### Subtheme 1.1: Diagnosis—An unknown fight

Diagnosis for LSDs is often long and uncertain, explored specifically in MPS and Pompe^78^, ^81^, ^83^: ‘It was a really, really long time from first ever hearing anything to getting any kind of real diagnosis’.^83^ (p. 1191). For some, the wait to receive a diagnosis was stressful and the rarity of the conditions often left families feeling unheard and in search of answers.^81^ Although an eventual diagnosis provided a sense of relief, frustration owing to the lack of information and guidance available ensued, described as: ‘walking in the dark’,^84^ (p. 2433).

The mode of diagnosis for many families was deemed poorly managed—received by families over the telephone by professional who did not have any knowledge of the condition. This exacerbated the distress associated with this time: ‘Over the phone it was horrendous for the reason being I had no support around me’,^78^ (p. 11).

###### Subtheme 1.2: Treatment and an unknown future

Treatment offered to children was embroiled with uncertainty, often experimental with an unknown outcome.^75^, ^78^, ^83^: ‘They are learning as they go. Every baby is almost like an experiment’,^83^ (p. 1192).

Pertinent questions for families across all LSD types were how long the treatment would help for,^75^ the ambiguity of the future and the progression of the condition. Increased worry associated with uncertainty was not uncommon,^48^, ^69^, ^75^, ^77^, ^80^, ^81^, ^83^, ^86^ increasing hypervigilance for symptoms: ‘… Any parent who is given a diagnosis of their child having a life‐threatening condition, a terminal condition, of course, your world is going to come crashing down because you do not know what is ahead of you’^78^ (p. 9).

Thinking about the future harboured a number of fears for families, particularly when accompanied by a loss of function and an increased reliance on care. For some, this was a signal to progression and the devasting idea of losing their child, resulting in recurring grief^75^, ^77^, ^80^: ‘I think mostly about his future (crying)’^75^ (p. 145).

##### Theme 2: All‐encompassing impact

It became evident that parenting a child with a LSD had an all‐encompassing impact on parents' lives.

###### Subtheme 2.1: Physical care demands

Receiving the diagnosis of a LSD is experienced as life‐changing with the physical care needs described as ‘continual’, supporting activities of daily living, medication administration and special diets. Managing care needs are time consuming, but also physically exerting and disturb sleep accounting for increased exhaustion.^58^, ^67^, ^71^, ^73^, ^74^, ^75^, ^77^, ^78^, ^80^, ^86^

The progression of LSDs and associated loss of function often leads to a higher reliance on physical care needs, explored widely within MPS.^71^, ^74^, ^75^, ^77^, ^80^ Care needs are ever changing, requiring ongoing adaptations^67^, ^71^, ^74^, ^78^: ‘It changes completely and it is very hard to realise that you have to change with our child … Yes, the condition is probably less mentally draining now and more physically challenging’,^78^ (p. 10).

###### Subtheme 2.2: Behaviour

In parity with physical care, behavioural challenges were pertinent in increasing the burden on parents, explored specifically within the context of MPS, Batten disease and Cystinosis.^67^, ^71^, ^73^, ^74^, ^78^ Oscillations between mood and behaviour were described as challenging and demanding^73^ and frequently included aggressiveness, hyperactivity, lack of fear and temper tantrums.

Compared with peers, behaviours were felt to be of increased intensity, leading to frustration, a need for increased vigilance and a reluctance to partake in activities outside of the home, increasing isolation and the physical and emotional burden.^67^, ^71^, ^73^, ^74^, ^77^ One parent quoted: ‘How much longer can I put up with this? You were just at your wit's end, just thinking every time I go near you, even to put socks…she'd lash out (…) that's the most challenging’,^71^ (p. 989).

Behaviours expressed by the child were sometimes understood in the context of progression of the condition, as a result of a frustrated child unable to communicate their needs: ‘There are things she wants to do (…) and then that is when we have melt‐downs or hitting or biting’,^74^ (p. 7).

###### Subtheme 2.3: Loss

Concurrent losses were a common experience for many within different facets of families' lives: socially (and relationally), economically and physically. Social losses were frequently discussed, with families restricted by lack of time due to caregiving responsibilities.^67^, ^69^, ^73^, ^75^, ^77^ Such social losses lead to social isolation changes to social relationships. Marital relationships were also not without strain, with increased care demands altering the relationship dynamic, and different illness‐led beliefs resulting in conflict: ‘It is the 600‐pound‐gorilla in the room. It causes a lot of tension between us because we have different opinions on what my son is capable of’,^84^ (p. 2433).

A change in working circumstance were common, with the child's primary caregiver having to change or stop working completely to care for their child, contributing to a loss in family finances but also a loss to parental identity, their position within the family and their imagined future^67^, ^73^, ^75^, ^77^, ^82^, ^83^, ^86^: ‘I had to adjust to the transition [from] being a full‐time working mom, contributing half to the family's income, to a stay‐at‐home mom …’,^73^ (p. 80).

Witnessing the physical losses to their child's function was distressing for families.^71^, ^74^ Anticipatory grief was often felt by parents as the progression of the condition reminded families of the inevitable loss of their child, whilst concurrently grieving for the child they once were^71^, ^73^, ^83^: ‘When he is ill, the fragility of his health makes me sad and reminds me of the unthinkable, unbearable reality that we could lose him’,^73^ (p. 79).

###### Subtheme 2.3: Emotional wellbeing

Parenting a child with a LSD had a clear impact on psychological and emotional wellbeing. Variations of increased worry, low mood and sadness were frequently iterated across LSDs.^67^, ^68^, ^69^, ^71^, ^73^, ^75^, ^77^, ^78^, ^83^, ^86^ Increased worry was as persistent for some parents as associated care needs: ‘All day long he is what I'm thinking about. I get very stressed out sometimes, to the point where I just what to cry all day long’,^73^ (p. 79). Alongside the devastation felt in relation to the diagnosis of a LSD, admissions of guilt associated with the impact of the condition on the child's future were also expressed.^73^, ^78^, ^81^, ^82^, ^83^

The associated physical and behavioural symptoms placed a significant burden on emotional wellbeing. Families often felt that they felt powerless, lacking control over the condition,^71^, ^73^, ^78^, ^81^ exacerbated by the progressive nature of the condition, increasing parental worry and distress^71^: ‘It is a constant worry … and you just feel like your life is trapped’,^78^ (p. 7).

##### Theme 3: Finding a way forward

A number of studies demonstrated how parents were learning to adjust to life with a diagnosis of a LSD and find a way forward with the condition.

###### Subtheme 3.1: A new reality

Adjusting to a new reality in light of living with a LSD left families habituating to a new way of living; establishing new routines and treatment regimens which soon become ‘normal’^73^, ^83^: ‘I feel like that first year was probably the hardest because it was the first time for everything (…) once you get through the first of everything the rest of it becomes a little easier because you have already experienced it. So, now our day to day is just a normal day to day’,^83^ (p. 1193).

Seeking support from other families with shared experiences and challenges, was found to be beneficial in adjusting to a new way of living,^68^, ^78^, ^83^, ^86^ providing families with a broader perspective of their child's condition and allowing them to feel helpful.^78^, ^83^, ^86^

###### Subtheme 3.2: A positive outlook

Focusing on the present was a key coping mechanism for families. Facilitated by the child's current state of wellness, this allowed families to remain positive^73^, ^78^, ^83^, ^86^: ‘You just kind of go down a spiral of what ifs and further and further, until finally you have to say ‘yes, but right now she is ok”,^83^ (p. 1191).

Contrasting with the burden felt by many, some families were able to focus on how the diagnosis had enriched their lives. Having a child with a LSD lead to narrowing families' focus toward their values^69^, ^73^, ^78^, ^83^, ^86^: ‘Instead of dwelling on any negative aspects associated with a chronic disease, I tried to help our family live a life of happy and meaningful moments’,^73^ (p. 80).

#### 3.3.3 Overall synthesis of findings

Overall synthesis of quantitative and qualitative data allowed for the relationships between factors to be summarised (see Figure 1). Combined, quantitative and qualitative data demonstrated that the physical care demands, and behavioural concerns related to the condition can all adversely influence parents' psychological and social outcomes. Physical and behavioural symptoms of different LSDs were significant relenting factors, highlighted by both quantitative and qualitative data, which qualitative data explained led to psychological distress and lower quality of life, and poorer social wellbeing due to significant lifestyle changes impacting on relationships, ability to work and parents' social lives. The uncertainty surrounding the conditions and the progressive and life‐limiting nature of LSDs were entangled with psychological distress, particularly grief and anxiety. Shared connections with other families who can relate to the difficulties that families face, maintaining hope and positivity and focusing on the present wellness of their child provided families with some coping mechanisms. These coping skills allowed parents to establish a new reality for their families in the midst of the life‐changing challenges which they faced. The extent to which such coping skills moderate the impact of parenting a child with a LSD remains unclear from the data.

## 4 DISCUSSION

This novel and substantive review demonstrates the substantial impact that parenting a child with a LSD has on the psychosocial functioning of parents. This is the first review of its kind to highlight and synthesise the significant impact and inherent challenges that parenting a child with a LSD brings. It established clear similarities of the challenges that parents faced despite heterogenous LSD diagnoses, aligning with challenges which are commonly recognised more widely in the chronic disease literature. However, the type of LSD can manifest particular difficulties, owing to the physical and behavioural symptoms which incidentally increased caregiver burden and adversely impacted physical, emotional and social wellbeing.

When considering the time that parents spend caring for their child due to the physical and behavioural demands, the disruption to parents' lives is unsurprising and often continues into adulthood. Therefore, parents can face long‐term losses within their lives, impacting on income, career and work opportunities, relationships, and home‐life. Such losses have been observed in other chronic conditions^88^ and IMDS, such as Urea Cycle disorders.^89^ Subsequently, the current review found that progression of the condition is important to take into consideration within both loss and the notion of uncertainty. Uncertainty was overwhelmingly experienced by parents across all LSDs and is a common finding within rare conditions. Research has shown increased uncertainty in chronic paediatric conditions results in poorer maternal mental health amongst mothers of children.^90^

Despite such a cumulative impact, parents demonstrated ways to cope by identifying positive aspects of parenting a child with a LSD a finding a new reality which existed for them, valuing support from other families affected by a LSD. In parents of children with physical disabilities, seeking support from other parents has also been found to be common, providing parents with experience‐based knowledge which they desired.^91^

### 4.1 Clinical and research implications

Adjusting to the diagnosis of a rare childhood condition can be a challenge for parents, which unmet psychosocial needs are likely to hinder.^92^ Parental adjustment is an important resource for children's adjustment,^93^ whilst increased psychological distress in parents is associated with poorer paediatric outcomes.^94^ The findings from this review highlight important implications for specialist services. Currently, in the United Kingdom, NHS England's service contract for children with LSDs only recognises the need for psychology within the context of neuropsychology for Gaucher's disease and ‘severe behavioural disturbance’ in MPS.^95^ This example of a service contract fails to recognise the significant challenges and support needs required for families. Increased support should be carefully considered within service specifications, with service designs aiming to increase psychological service provision which validates the widespread level of psychological challenges within this client group. Improving communication, that is, delivery of a diagnosis and accompanying complex information, is an important aspect of care. As outlined by Burgard,^96^ this is essential to support the life‐long dynamic care and treatment needs often executed by families at home. Further research needs to determine how accessing resources and support helps parents of children with LSDs whilst also establishing the type of support parents would find helpful.

### 4.2 Strengths and limitations

The integrative nature of this review was a strength which has allowed for an in‐depth understanding of the psychosocial implications of parenting a child with a LSD. Steps were taken to ensure scientific rigour and transparency of the review process and to reduce bias within study screening, selection, data extraction and quality appraisal.

Despite such strengths, there are potential limitations which need to be considered, such as publication and language biases. However, such exclusions ensured a baseline of scientific rigour. As only three studies were excluded on the basis of language (eg, Refs. 97, 98, 99), their exclusion was not considered to affect the overall analysis. Furthermore, no studies were excluded based on their quality. Finally, the approach used to assess quality was rigorous and transparent and highlighted the need for more good quality studies.

## 5 CONCLUSION

This review has highlighted the lack of quality research in understanding how different LSDs influence parental experiences. However, our synthesis demonstrated the significant psychosocial impact that having a child with a LSD can have on parents, potentially resulting in unmet parental needs exacerbated by physical and behavioural care demands. There is a need for increased support and monitoring of parents' adjustment and mental health. Parental adjustment to diagnoses is key to children's adjustment and outcomes and therefore designing a service to target the challenges which parents experience is imperative.

## CONFLICT OF INTEREST

The authors declare no conflicts of interest.

## AUTHOR CONTRIBUTION

Anja Wittkowski, Sadie Hassall and Debbie Michelle Smith developed the review concept. All authors contributed to the study design. Sadie Hassall completed the systematic search and extraction of data. Extracted data were checked by Anja Wittkowski and Debbie Michelle Smith. The synthesis of quantitative data was completed by Sadie Hassall under the supervision of Anja Wittkowski and Debbie Michelle Smith. Sadie Hassall and Debbie Michelle Smith completed the thematic analysis of qualitative data. Sadie Hassall completed the quality appraisal under the supervision of Stewart Rust, with the overall review process being overseen by Anja Wittkowski. Sadie Hassall drafted the review and Anja Wittkowski, Debbie Michelle Smith and Stewart Rust provided critical revisions. All authors have approved the final version of the review for submission. Anja Wittkowski acts as a guarantor for the article.

## ETHICS STATEMENT

This article does not contain any studies with human or animal subjects performed by the any of the authors.

## Supporting information


**Table S1** Search strategy and terms.
**Figure S1** PRISMA flow diagram outlining systematic process.
**Table S2** Data extraction table demonstrating relevant findings from studies grouped according to LSD type (most frequently occurring to least).
**Table S3** Methodological quality and risk of bias appraisal table.

## Data Availability

Data sharing is not applicable to this article as no new data were created or analysed in this study.
