# Supplementary material for: A systematic review and integrative sequential explanatory narrative synthesis: The psychosocial impact of parenting a child with a lysosomal storage disorder
Source: J Inherit Metab Dis. 2022 Feb 24;45(3):406–16. doi: 10.1002/jimd.12482 (PMC9305282; doi:10.1002/jimd.12482)
Supplement: Supplementary file 1 — Table S1 Search strategy and terms. Figure S1 PRISMA flow diagram outlining systematic process. Table S2 Data extraction table demonstrating relevant findings from studies grouped according to LSD type (most frequently occurring to least). Table S3 Methodological quality and risk of bias appraisal table. [file JIMD-45-406-s001.docx]

**Supplementary Table S1: Search strategy and terms**

| **Search strategy** | **PICO framework** | **Search terms** |
| --- | --- | --- |
|  | **P - population** | Parent* OR family OR, mother OR father OR caregiver* |
|  | **I - intervention** | Not applicable |
|  | **C - comparison** | Fabry disease OR Gaucher disease OR Hurler Scheie OR Scheie syndrome OR Hurler syndrome OR Hunter syndrome OR Sanfilippo syndrome OR Sly disease OR Schindler disease OR Morquio syndrome OR Maroteaux-lamy syndrome OR Pompe disease OR alpha mannosidosis OR Tay-Sachs disease OR Niemann Pick disease OR Batten disease OR cystinosis OR Lysosomal Storage Disorder OR inherited metabolic disease OR inborn error of metabolism,OR Mucopolysaccharidosis Type I* OR Mucopolysaccharidosis Type V* OR MPS I* OR MPS V* OR Acid Lipase Deficiency OR Wolman disease OR CLN2 OR Janksy-Bielschowsky disease |
|  | **O - outcome** | Quality of life OR QOL OR well-being OR wellbeing OR adjustment OR adaptation OR acceptance OR adaptive OR psychological OR psychiatric OR psychosocial OR emotional OR mental health OR social OR mental disorder OR stress OR depression OR anxiety OR coping OR resilience |
|  | **1 AND 2 AND 3** |  |

Papers identified through electronic searching n= 2595

PschInfo (n=54); Embase (n=1490); Medline (n=74); CINAHL (n=82); Web of Science (n=895)

Identification

Duplicates Removed n= 268

Papers screened based on title and abstract n = 2327

Screening

Papers excluded based on title and abstract n=2174

Eligibility

Full text citations reviewed for inclusion n=153

Papers excluded based on:

- Full text not available n= 47
- Not peer reviewed/ dissertation n=5
- Not available in English n= 3
- No or undistinguishable LSD n=17
- No parent measure n=50

n=122

Citations included from hand searching n= 7

Inclusion

Mixed study citation included n=5

Qualitative citations included n=10

Quantitative citations included n=23

Total studies included n=38

***Supplementary Figure S1: PRISMA flow diagram outlining systematic process***

**Supplementary Table S2**

**Data extraction table demonstrating relevant findings from studies grouped according to LSD type (most frequently occurring to least)**

| **Quantitative studies (n=23)** | | | | |
| --- | --- | --- | --- | --- |
| **Reference and country** | **Study design** | **Relevant measures** | **Sample demographics** | **Relevant findings** |
| Hoffman, Hoffman, Kunzmann & Ries (2020) (24)  Germany | Cross-sectional survey | Study specific questionnaire | MPS I (Hurler syndrome) n=7, MPS IS (Scheie) n=1, MPS IH: n=7,  MPS II (Hunter syndrome) n=8 (severe subtype: n=5; subtype unknown: n=2; mild subtype n=1) MPS III (Sanfilippo syndrome): n=18 (MPS IIIA: n=11; MPS IIIB: n=7). n=18 male children, mean age 8.7 years.  Questionnaires completed by: mother (n=28), both parents (n=3), father, grandfather or sister (n=1 respectively).  No parent/ caregiver demographics reported.  No comparator/reference group. | Symptoms associated with severe parenting stress according to scores of 4-5 on Visual Analogue Scale (VAS):   - Agitation - Aggression - Sleep disturbance   Symptoms associated with moderate parenting stress according to scores of 2-3 on VAS:   - Hyperactivity - Repeated behaviour - Unusual affect - Apathy   Frequency of symptoms and level of parenting stress varied with MPS type. |
| Conner, Cook, Fernandez, Rascati & Rangel-Miller (2019a) (25)  USA | Cross-sectional survey | Modified BURQOL-RD (23) | MPS I (Hurler syndrome)  n=32 parents  average age of child 9.4 years.  No parent demographics reported.  No comparator/ reference group. | Caregiver burden post HSCT in relation to work:   - 23% of parents reduced work hours - 69% of parents had difficulty completing work tasks due to their child’s health and treatment - Mean of 8.5 hours per day spent on caring. |
| Conner, Cook, Fernandez, Rascati &Rangel-Miller (2019b) (26)  USA | Cross-sectional survey | Modified BURQOL-RD (23) | MPS II (Hunter syndrome) parent caregivers of 50 children with a mean age of 9.4 years.  No parent demographics reported.  No comparator/ reference group. | Caregiver burden for children receiving ERT in relation to work:   - 25 parents worked part-time, 11 were employed full time and 33 unemployed.   Hours spent caring varied by age:   - Parents of children <10 average of 13 hours a day caring - 8.8 hours a day for child >10. |
| Morrison et al. (2019) (27)  Germany, Netherlands, Spain and Turkey | Cross-sectional | Study specific questionnaire | MPS VII (Sly disease)  n=12 parent/caregivers of 13 children (mean age of children 17.1 years).  No parent demographics reported.  No comparator/ reference group. | - 46.2% of parents had to reduce work hours - 30.8% stopped work to care for their child - 53.8% of MPS VII patients required support with daily activities. 30.8% required more support throughout the day - Two families had to move house and three had to make adaptations to their living environment. |
| Conijn, Nijmeijer, van Oers, Wijburg & Haverman (2018) (28)  Netherlands | Cross-sectional | Hospital Anxiety Depression rating Scale (HADS; 29)  Distress Thermometer for parents (DT; 30)  Self-rating scale for posttraumatic stress disorders (SRS-PTSD; 31) | MPS III (Sanfilippo syndrome) n=45 parents of 34 patients (mean patient age 19.8, range 5-38).  26 Mothers: mean age 48.7 years, educational status (N) - low (7), intermediate (9) and high (10), marital status (N) – married/ live together (23) single/ separated (3)  19 Fathers: mean age 50.1 years, educational status (N) - low (2), intermediate (7) and high (10), marital status (N) – married/ live together (18) single/ separated (1)  Reference group: scores compared to general population. | - 50 % of mothers met clinically relevant score for anxiety; 34.6% for depression - Fathers were more likely to meet clinical levels for depression than the reference group - 84.6% of mothers and 68.4% of fathers demonstrated clinically relevant distress - PTSD symptoms higher in both parents (26.9% mothers and 15% of fathers). |
| Pentek et al (2016) (23)  Bulgaria, France, Germany, Hungary, Italy, Spain and Sweden | Cross-sectional | Social Economic Burden and Health Related Quality of Life in patients with Rare diseases (BURQOL-RD; 23)  Zarit Burden Interview (ZBI; 32),  EQ-5D (33) | MPS – varied but does not state which type  120 patients with MPS – 74 children and 49 adults (mean age 15.4)  66 non-professional caregivers (65 parents and 1 partner/ ‘other’)  No parent/caregiver demographics reported.  No reference/ comparator group. | - Mean informal care time 51.3 hours per week (51.0, SD 64.4 for children; 51.8, SD 68.2 for adults) - Burden ranged from 20.5 in French sample (little burden) to 54 in the Sweden sample (moderate to severe burden) - Burden reported from parents in Germany, Italy and Spain fell within the mild to moderate range. |
| Lehtonen, Rust, Jones, Brown & Hare (2016) (34)  UK | Cross-sectional | Parent Stress Index short form (PSI-SF; 35) | MPS 1H (Hurlers syndrome)  21 patients/ children (mean child age 9 years 7 months)  19 parents/ families – no demographic data reported.  Reference group: general population. | - Five parents clinically elevated range for stress - Raw scores not significantly higher than the general population. |
| Guarany et al. (2015) (36)  Brazil | Cross-sectional | The World Health Organization Quality of Life Assessment (WHOQOL-BREF; 37) | MPS I (n=1), MPS II (n=1 severe, n=2 attenuated), MPS III B (n=3), MPS IV-A (Morquio A; n=4)  MPS VI (n=1)  Median age of child/ patient 16.5 years  11 mothers: median age 35 years, range 29-43.  No comparator/ reference group. | - QOL associated with severity of condition - Lower scores on psychological, social and environmental domains - Most scores fall between low- moderate range. |
| Guffon et al. (2015) (38)  France | Cross-sectional | Study specific questionnaire | MPS II (severe n=36, attenuated n=16), mean age 12.4.  44 parent respondents – no demographics reported.  Reference group: reference population for child scores. | - 20 parents had to reorganise working hours due to care of child - In 10 sets of parents one parent had to stop work - Mothers affected more frequently. |
| Michalik (2014) (39)  Czech Republic | Cross-sectional | Study specific questionnaire | MPS (does not specify type)  33 Parents of children with MPS, cystic fibrosis (n=113), achondroplasia (n=47), metabolic disease (n=59), spinal Musculo dystrophy (n=46).  No parent/child demographics reported.  Comparator groups: Cystic Fibrosis, Spinal Muscular Atrophy, metabolic disorders and Achondroplasis. | - MPS group reported highest economic deterioration - Increased deterioration in family relationships and social life - Poorer evaluation of caregiving - Increased fears about the future. |
| Hendriksz et al. (2014) (40)  Brazil, Columbia, Germany, Spain, and Turkey | Cross-sectional | Study specific questionnaire  ZBI (32) | MPS IVa (n= 56 patients, mean age 14.1 years, range 1-46)  56 parent caregivers (mean age 42.6, range 16-71).  No comparator/ reference group. | - Physical health difficulties in parents:   - 68% reported back pain   - 22% reported gastrointestinal problems   - 46% reported poor sleep   - 70% reported increased stress - Mental health difficulties:   - 46% stated that they felt burdened   - 41% reported symptoms of anxiety   - 43% reported symptoms of depression - Financial difficulties (high unemployment):   - 46% of caregivers of children were employed.   - Of employed parents 11% worked full time   - Increased costs for care associated with moving/adapting house or buying an adapted car or a wheelchair. |
| Buraczewska, O’Leary, Walsh, Monavari & Crushell (2013) (41)  Ireland | Child/ patient: observational retrospective cohort study (single arm) Parent: cross-sectional survey | Study specific questionnaire | MPS Type II  9 families of children age range: 3.5-14 years.  No parent demographics reported.  No comparator/ reference group. | - Six families admitted experiencing psychosocial difficulties due to weekly hospital visits for ERT - Adverse effect on parental employment in 8 families (at least one parent not working/ reducing availability for work). |
| Grant et al. (2013) (42)  UK | Cross-sectional survey | The Paediatric Inventory for Parents (PIP; 43)  General Health Questionnaire (GHQ-12; 44)  Resilience Scale for Adults (RSA; 45) | MPS III (n=16 children), mean child age 8.63, range 2-15 years.  23 parents of children with MPS III compared to 23 parents of children with an intellectual disability.  16 mothers, 7 fathers. Mean age 35.8 (range, 23-49).  Ethnicity: British (n=13), European (n=2), Pakistani (n=7), unknown (n=1).  Education: GCSE (n=7), A-levels (n=5), Undergraduate (n=2), postgraduate (n=2), other (n=1), unknown (n=6).  Comparator 30: parents of children with an ‘intellectual disability’. | - Parents scored above the clinical cut off for anxiety and depression – not significantly different to comparison group - PIP score significantly higher than mothers within oncology services - Social resources were a significant protective factor – significant other provided more support than others. |
| Raluy-Callado, Chen, Whiteman, Fang & Wiklund (2013) (46)  USA, UK, Brazil and Germany | Cross- sectional survey | Child Health Questionnaire -Parent Form (CHQPF-50; 47) | MPS II (n=96 ‘patients’), mean age 14.2 years.  96 parents – no demographic data reported.    Reference group: published data in paediatric literature. | - Poorer family cohesion scores compared to other paediatric groups - Parental impact on emotional scale was lower than a cohort with asthma. |
| Ucar et al. (2013) (48)  Turkey | Cross over case series | Hamilton Anxiety Scale (HAM-A; 49)  Beck Depression Inventory (BDI; 50) | MPS IIIA (n= 12 ‘patients’/children), mean age 5.7 years, range 2.5 - 10.6 years.  12 mothers – no demographics reported.  No comparator/ reference group. | - Depression scores decreased with treatment for child’s behaviour although scores still fell within the moderate range on the BDI - Mild to moderate anxiety ratings before and after treatment. |
| Kuratsubo et al. (2009) (51)  Japan | Cross-sectional survey | General Health Questionnaire 60 (GHQ-60; 44)  State-Trait Anxiety Inventory (STAI; 52) | MPS II (n=10 ‘patients’), mean age 23.4, range 39-13 years.  6 fathers and 5 mothers – no demographic data reported.  Reference group: unspecified reference group from previously published data. | - Relatively higher GHQ-60 scores - Relatively higher levels of state and trait anxiety than the general Japanese population. |
| Bax & Coville (1995) (53)  UK & non-UK (not specified) | Cross-sectional survey | Study specific questionnaire | MPS I (n=63), MPS II (n=54), MPS III (n=106) and MPS IV (n=35). Age range of children 0-14.  258 parents/ families – no demographic data reported.  No comparator/ reference group. | - Four main areas of concern established for parents:   - Prognosis and multiple health problems   - Behaviour and lack of communication   - Distress regarding lack of communication   - Anxieties around having more children. - Parents reported not being able to find help for these concerns but received support from:   - MPS society newsletters   - Conferences   - Parental network. |
| Nidiffer & Kelly (1983) (54)  USA | Cross-sectional survey | Study specific questionnaire | MPS III (n=30), mean age 10.5 years, range 11months-22 years, median age 8.8 years.  30 parents – no demographic data reported.  No comparator/ reference group. | - 86% of parents reported marital stress - Reduced time for social and personal activities - Parents reported feeling anger (4 parents), helpless (6 parents) and guilt (3 parents) - Changes to parents’ lives were reported in the context of holidays (17 parents), fewer friends (13 parents) and lack of time for family and social activities (12 parents) - Embarrassment to go out in public was reported by eight parents. - Support needs highlighted by parents:   - Parenting group (exchange information/ emotional support)   - Education for professionals and others about the disease |
| Adams, Mink & University of Rochester (2013) (55)  USA | Retrospective cohort study | Pediatric Quality of Life Inventory (PEDS QoL) Family Impact Module (56) | Juvenile neuronal ceroid lipofuscinosis (Batten disease)  Initial sample: n=44 ‘patients’ and parents respectively, mean age of patients =14.0 years  Follow-up sample: n=51 patients and parents respectively, mean age =16.3 years  No demographic data reported for parents.  No comparator/ reference group. | - Correlation between PEDS QoL family impact score and severity of disease - Behaviour problems significantly associated with reduced parental emotional, social and cognitive wellbeing - Problems in family relationships negatively associated with adaptive function of the child. |
| Labbe, Lopez, Murphy & O’Brien (2002) (57)  USA | Cross-sectional survey | The Life Orientation Test (58),  Caregiver Reaction Assessment (59),  Symptom Checklist-90-R (60),  The Family Environment Scale (FES; 61) | Batten disease (median age of children 13 years).  28 mothers, 4 fathers: median age 39 years, mean years of education 13.6.  Comparison group: 20 mothers of children with other neurological conditions). | - Higher mean depression, anxiety and Negative Impact on Health and Schedule compared to the comparison group - FES indicated no mean differences with conflict or cohesion. |
| Cozart, Augustine, Mink, Thatcher & Adams (2017) (62)  USA | Cross-sectional survey | Study specific questionnaire | Batten disease  29 parents (n=22 mothers,  n=7 fathers).  N=26 married, n=2 unmarried/ single, n=1 long-term relationship. Parents average age 49.6 years.  No patient/ child demographics reported.  Reference group: general population and parents of children with chronic but non-degenerative conditions. | - 28% reported marital distress (DAS score of <97) - higher than the general population and parents of children with chronic, non-degenerative illnesses - Parents sought support from specialists, the internet, and other parents of children with Batten disease - 93% of parents sought additional support and information regarding Batten disease using social media or websites - Support from other parents was the most endorsed form if support. |
| Kanters, van der Ploeg, Brouwer & Hakkaart (2013) (63)  Netherlands | Cross-sectional survey | CarerQOL(64),  Self-rated burden scale (65) | Pompe disease (n=67 ‘patients’: <18 (n=18)  Caregivers (n=66): 45 partners, 19 parents, 2 ‘other’  No demographic data reported.  No comparator/ reference group. | - Parent carers (‘patients’ below the age of 18) reported higher burden - No reports of well-being from providing care in comparison to carers of adults - 31 hours per week average care for children. |
| Adam et al. (2019) (66)  UK | Cross-sectional survey | Hospital Anxiety Depression rating Scale (HADS; 49)  Caregiver Strain Index (CSI; 67) | Alpha-mannosidosis (n=9 parent/caregiver)  Age range of patient 7-37 years (6-11 (n=1), 12-17 (n-3), >18 n=5).  No parent/ caregiver demographics reported.  No comparator/ reference group. | - Wheelchair dependence impacted negatively on quality of life. - 56% borderline/ abnormal scores in at least one subscale (anxiety/depression) - High level of stress related to care provision   Stress and anxiety impacted on family relationships and dynamics   - 16.6h per day spent caregiving - Seven out of nine carers were unable to work full-time. |
| **Mixed methods studies (n=5)** | | | | |
| Shapiro et al. (2019) (68)  USA, UK, Spain, Turkey, Japan, Argentina, Brazil, Canada, and Portugal | Quantitative data: Cross-sectional survey.  Qualitative analysis: further details are not provided | Qualitative interview/ survey | MPS IIIB (Sanfilippo B: Average age of children of parents 9 years, range 4-50 years)  26 parents  5 clinical experts  46 clinicians and patient advocates.  Parent demographics not reported.  No comparator/ reference group. | - Caregiver burden related to:   - emotional impact of coping with the disease   - associated behaviour (e.g., aggressiveness),   - communication difficulties - Burden of care impacted on the parent’s ability to work, their quality of life and the family system as a whole - Daily responsibilities leave caregivers with little time for other things, restricting families from doing ‘normal’ activities (differ depending on age of child, symptoms and behaviour) - Psychological stress and physical exhaustion as a result of continued care burden results in depression and isolation - reinforced by disease progression and loss of acquired skill. |
| Schadewald, Kimball and Ou (2017) (69)  USA | Cross-sectional survey  Qualitative analysis: further details are not provided. | Pediatric Inventory for Parents (PIP; 43) and qualitative questions | MPS I (n=21), MPS II (n=19), MPS IIIA (n=14), MPS IIIB (n=9), MPS IVA; n=7) or MPS VI (Maroteaux-Lamy syndrome; n=9) – total children (n=100) - further demographic data not reported.  93 parents (for 11 participants data was not analysed). Age range 25-64. Ethnicity: Caucasian (n=62), African (n=4), Asian (n=3).  Education: undergraduate degree (n=24), graduate degree (n=11), high school (n=11), associate degree (n=8).  Marital status: married (n-67), divorced/ single (n=6), separated (n=3).  16 parents had 2 children with MPS.  No comparator/ reference group. | - Coping strategies:   - Increased psychosocial support increased caregiver acceptance, but not positive coping strategies   - Education on behavioural changes related to the diagnosis and interventions reduced denial but guidance on what to expect from the disease process increased denial. - Stress:   - Emotional distress contributing frequently and significantly to parenting stress   - Psychosocial support had no influence on stress levels. - Qualitative findings:   - 21% of caregivers found online support and contact with other families as the most helpful   - 36.5% would like more emotional support services   - 53.8% would like more information on community services   - Parents reported feeling lonely, sad, and concerned about the lack of support and information. |
| Needham et al. (2013) (70)  UK, Canada, Ireland, Australia | Cross-cross sectional survey  Qualitative analysis: Summative content analysis (71) | PEDSQoL Family Impact Module (56)  Supplemental questionnaire (Qualitative) | MPS II (n=73, age range of 68 patient 2-21, 5 patients >21. Ethnicity of patients: Caucasian (n=60), Asian/ Pacific Islander (n=5), Latino (n=2), other (n=6). 22 patients had attenuated MPS II, 29 had severe MPS II and 22 were of unknown classification.  73 parents/caregiver – demographic data not reported.  Reference group: ‘patients’/ parents of healthy children. | - Greater overall family impact than other paediatric groups. - Severity of condition related to decreased QOL for caregivers. - Qualitative themes:   - Fear of child’s death/ life expectancy   - Worry in relation to child’s pain and future functioning   - Coping   - Social isolation. |
| Malcolm, Forbat, Anderson, Gibson & Hain (2011) (72)  UK | Prospective cohort study  Qualitative analysis: Grounded Theory (73) | Symptom diary and qualitative interviews | MPS I (n=1, age 2), MPS III (n=15, age range 5-21, median age 9), MPS IVA (n=1, age 8), and Batten disease (n=9, age range 2.5-18, median age 5).  23 parents – no demographic date reported.  No non-LSD reference/ comparator group. | - The universality of behavioural symptoms and the resultant family exhaustion   - Coping and emotional impact with behavioural symptoms of disease (particularly MPS III)   - Poor sleep   - Emotional significance of disease progression.   - Impact on all areas of family life. - The emotional impact on families of symptoms that are difficult to control   - Increased parental anxiety and lack of control due to physical health of child Grief reaction related to deterioration and loss of skill in children – particularly in relation to Batten disease. |
| Spilkin & Ballantyne (2007) (74)  Location not specified | Cross-sectional survey  Qualitative analysis: further details are not provided | Cystinosis Behaviour Questionnaire – study specific questionnaire | Cystinosis (n=63 children; mean age 8.5 years)  63 primary/secondary caregivers completed the questionnaire.  Primary caregiver: mother (n=58), father (n=4), or grandfather (n=1).  Secondary caregiver: father (n=35), stepfather (n=2), mother (n=1),  grandmother (n=2), grandfather (n=1),  aunt (n=1), or sibling (n=1).  Comparator group: Parents of healthy children. | - Difficult behaviours within their children - Parents reported a number of strengths but also family stressors:   - Worrying/ stress   - Pain and sadness that their child’s illness (thoughts about losing their)   - Impact on economic factors due to not being able to work fulltime (appointments, child’s sickness and poor sleep)   - Impact on social wellbeing, taking on too much responsibility, and family life revolving around the child - Positive aspects of coping:   - Not taking things for granted, having a better career and family balance, and becoming aware of the true values and happiness in life. |
| **Qualitative studies (n=10)** | | | | |
| Porter et al., (2020) (75)  USA | Thematic analysis (21) | Focus groups | MPS III Type A (n=17), Type B (n=6) and Type C (n=2). Median age of child = 8 (range 4-36 years)  Parents (24 biological parents, 1 step-parent). Median age 38 (range 29-65 years). Females (m=19), males (n=6). Ethnic background: Caucasian (n=23), more than one ethnicity (n=2). | - Symptoms associated with communication difficulties and behaviours were challenging - identified as carrying the highest burden for parents - Communication difficulties led to child frustration and behaviour problems and parents found it difficult to meet the evolving needs of the child as the condition progressed - Difficult to manage behaviours during periods of progression were prominent in relation to caregiver burden. Parents felt that they had to be vigilant and watch child at all times to maintain their and others’ safety - Sleep disturbance leads to difficulties in parent’s day-to -day functioning - Increased caregiver time due to physical care demands. |
| Zengin, Yayan & Akinci (2020) (76)  Turkey | Colaizzi's phenomenological method (77) | Semi-structured Interview | MPS II (n=4), MPS IV (n=5), MPS VI (n=1) (mean age 8.8).  Parents (4 mothers and 4 fathers). Mean age of mothers 32.6, mean age of fathers 36. | - Physical difficulties associated with care of children (fatigue) - Difficulties within parents’ social life (emotional impact of comparison to typically developing children and behavioural concerns in social situations) - Concerns in relation to fearing losing their child, uncertainty about the future and treatment and fear of separation from their child - Hope and religion helped parents to cope. |
| Harrington et al. (2019) (78)    USA, Canada, UK, Mexico, Spain | Content analysis (71) | Interviews | MPS II (n=22 caregivers) and MPS IIIA (n=8 caregivers)  Mean age range 37.9-41.3  Female/ mother (n=27)  Ethnic background: Black (n=1), white (n= 19), mixed African/white (n=1), not reported (n=9).  Marital status: Married (n=20), Single (n=1), other (n=1).  Employment status: full time/ part time (n=18), homemaker (n=9), unemployed/ retired (n=3)  Education level: Primary school (n=2), Secondary school (n=4), Associates degree/ vocational (n=4), college (n=7), university degree (n=7), postgraduate degree (n=6). | - Social functioning: - Behaviour problems of child lead to difficulties spending time doing family activities/ as a couple. Limited social interactions lead to isolation. - Emotional/ psychological functioning: - Behavioural difficulties of the child impacting emotional well-being. Fears about the future (anxiety) and grief (hopelessness, frustration and helplessness). - Progression of condition leading to depression - Physical functioning: - Fatigue in relation to care, behaviour and emotional burden. - Daily functioning: - Interrupted work (care/ medical appointments), inability to leave child unattended. |
| Somanadhan & Larkin (2016) (79)  Ireland | Thematic analysis (80) | Interview | MPS I, II, III and VI (children/ ‘patients’ aged 6 months- 22 years).  8 parents – demographic data not reported | - Living with MPS evokes strong emotions such as uncertainty and ambiguity - Parents describe their emotions as being confused, upset frustrated and devastated by the enormity of the debilitating and life-limiting nature of MPS - Families reported that marriages had become strained since the diagnosis - Long and uncertain route to diagnosis. The diagnosis itself led to anger, frustration, shock and devastation - Parents reported feeling worried as their child failed to catch up to their peers with the gap widening as they become older. - Parents reported feeling powerless with constant worry and stress about their child’s prognosis - Internet as helpful as a resource to communicate with other families - Parents explained trying to focus on the positives. |
| Soni-Jaiswal, Mercer, Jones, Bruce & Callery (2016) (81)  UK | Grounded theory (73) | Interview | MPS I (n=11 children, 5 Hurler, 6 attenuated, mean age 7 years, age range 6 months-16years)  11 families – no parent demographics reported. | - Parents expressed worry about the symptoms of MPS, their child’s future (not having an independent life) and their child’s wellbeing (burdened with anxiety and depression due to diagnosis). |
| de Ru, Bouwman, Wijburg & van Zweiten (2012) (82)  Netherlands | Qualitative analysis – further details are not provided | Interview | MPS IH (n=6), MPS IH/S (n=4), MPS I S (n=7), mean age 9, range 3-22 years.  17 parents – no demographic data reported | - Impact of delayed diagnosis on parents’ emotional wellbeing - Parents considered how early diagnosis would have impacted on initiation of treatment and ability to focus on the on the diagnosis. |
| Young & Harfer (1981) (83)  UK | Qualitative analysis – further details are not provided | Interview | MPS II  21 sets of parents (severe)  17 sets of parents (mild)  No demographic data reported. | - Severe MPS: - Almost all mothers experienced feelings of guilt - Most mothers had overcome confusion and denial about the condition, although several struggled with acceptance characterised by unrealistic future expectations - Most marriages consolidated by diagnosis. - Mild MPS - Most mothers were reported to appear distressed, ‘devoting’ much of their life to the care of the child - Three out of 12 marriages ended in divorce. |
| Pruniski, Lisi & Ali (2018) (84)  USA | Grounded Theory (73) | Interviews | Pompe disease – Late onset Pompe Disease (LOPD) and infantile onset Pompe Disease (IOPD), age range 6 months - 3 years.  9 mothers – 6 LOPD and 3 IOPD  All mothers Caucasian and married.  Employment: Homemaker (n=1), Employed (n=8).  Education level: High school (n=1), college (n=2), graduate (n=6). k | - Uncertainty due to initial diagnosis, the future and symptom onset and treatment initiation - Emotional reactions expressed as grief over the loss of the healthy child, sadness, gratefulness at being able to plan for the future, guilt, and loneliness/isolation - Impact on family dynamics – strain vs. positive impact - Caregiver burden was intensified by caring for other siblings, managing employment with medical appointments and having to change employment or reduce their work schedule leading to financial concerns - Coping: normalisation and giving the child a normal family life was key to coping, as well as developing a new normal and being proactive in advocacy within the Pompe community. |
| Henderson, Packman &Packman (2009) (85)    USA | Qualitative case study (86) | Interview | Niemann-Pick disease Type B (n=8 patients), mean age 28.4years, range 16.8-43 years.  9 parents (5 mothers, 4 fathers), mean age 45.4 years, range 38-53 years)  Education: High school diploma/ equivalent (n=4), 1-3 years college/ equivalent (n=5).  Marital status: Married/partner ((n=5), Divorced (n=4). | - Parents reflected on their experiences of diagnosis (frustration and feeling scared) - Parents reported grief following the diagnosis and worrying about the progression of the disease - Cause of tension due to differing opinions and balancing the needs of child with the diagnosis and other siblings. |
| Freedman (2013) (87)  Australia | Thematic Analysis (21) | Interview | MPS I (n=1 children), MPS II (n=3 children), Pompe (n=0), Gaucher (n=0), age range for children 10-15 years.  9 parents – MPS I (n=1), MPS II (n=5), Pompe (n=2), Gaucher (n=1)  No parent demographics reported. | - Impact on work (treatment and appointments) - Parents commonly worried about the future (uncertainty) - Parents tried not to focus on if the child will get worse/ things parents can’t control - Parents found speaking to other families was helpful. |

**Supplementary Table S3Methodological quality and risk of bias appraisal table**

| **Quantitative studies** | Framework | Aims | Setting | Sample size | Target sample | Procedure | Tools | Recruitment | Validity of tools (Quant) | Data Collection Fit (Quant) | Data Collection Fit (Qual) | Analysis fit | Analytical method | Analytical process(Qual) | User involvement | Strengths/ limitations | Score out of 42 (%) |
| --- | --- | --- | --- | --- | --- | --- | --- | --- | --- | --- | --- | --- | --- | --- | --- | --- | --- |
| Hoffman et al. (2020) | 1 | 2 | 3 | 0 | 2 | 3 | 2 | 2 | 0 | 2 |  | 2 | 2 |  | 2 | 3 | 26  (62%) |
| Conner et al. (2019a) | 2 | 3 | 3 | 0 | 2 | 2 | 1 | 2 | 0 | 2 |  | 2 | 0 |  | 0 | 2 | 21 (50%) |
| Conner et al. (2019b) | 1 | 3 | 3 | 0 | 2 | 2 | 1 | 2 | 0 | 2 |  | 2 | 0 |  | 0 | 2 | 20 (48%) |
| Morrison et al. (2019) | 0 | 2 | 3 | 0 | 2 | 3 | 1 | 2 | 0 | 2 |  | 2 | 1 |  | 0 | 2 | 20 (48%) |
| Conjin et al. (2018) | 1 | 3 | 3 | 0 | 2 | 2 | 3 | 3 | 3 | 3 |  | 2 | 1 |  | 0 | 2 | 26 (62%) |
| Pentek et al. (2016) | 1 | 3 | 1 | 0 | 2 | 2 | 3 | 1 | 1 | 3 |  | 2 | 0 |  | 0 | 2 | 21 (50%) |
| Lehtonen et al. (2016) | 2 | 3 | 2 | 0 | 1 | 2 | 2 | 2 | 1 | 3 |  | 3 | 2 |  | 0 | 1 | 24 (57%) |
| Guarany et al. (2015) | 3 | 3 | 3 | 0 | 2 | 2 | 0 | 2 | 0 | 2 |  | 1 | 0 |  | 0 | 1 | 19 (45%) |
| Guffon et al. (2015) | 1 | 3 | 3 | 0 | 2 | 2 | 2 | 3 | 0 | 2 |  | 3 | 1 |  | 0 | 2 | 24 (57%) |
| Michalik (2014) | 2 | 0 | 3 | 0 | 1 | 1 | 2 | 2 | 0 | 0 |  | 2 | 1 |  | 0 | 0 | 14 (33% |
| Hendriksz et al. (2014) | 1 | 3 | 3 | 0 | 2 | 3 | 2 | 1 | 1 | 3 |  | 1 | 1 |  | 3 | 1 | 25 (60%) |
| Buraczewska et al. (2013) | 0 | 3 | 3 | 0 | 1 | 1 | 0 | 0 | 0 | 2 |  | 0 | 0 |  | 0 | 0 | 10 (24%) |
| Grant et al. (2013) | 3 | 3 | 3 | 0 | 2 | 2 | 2 | 2 | 1 | 2 |  | 2 | 2 |  | 0 | 2 | 26 (62%) |
| Raluy-Callado et al. (2013) | 1 | 3 | 2 | 0 | 2 | 2 | 2 | 2 | 3 | 3 |  | 3 | 1 |  | 0 | 2 | 26 (62%) |
| Ucar et al. (2013) | 1 | 3 | 2 | 0 | 1 | 2 | 2 | 1 | 0 | 3 |  | 3 | 0 |  | 0 | 2 | 20 (48%) |
| Kuratsubo et al. (2009) | 1 | 3 | 2 | 0 | 2 | 2 | 0 | 1 | 0 | 2 |  | 2 | 0 |  | 0 | 0 | 15 (35%) |
| Bax & Colville (1995) | 1 | 1 | 2 | 0 | 2 | 2 | 0 | 2 | 2 | 2 |  | 0 | 0 |  | 0 | 2 | 16 (38%) |
| Nidiffer & Kelly (1983) | 1 | 3 | 2 | 0 | 1 | 2 | 1 | 2 | 2 | 2 |  | 2 | 0 |  | 0 | 1 | 19 (45%) |
| Adams & Mink (2013) | 1 | 1 | 3 | 0 | 3 | 1 | 1 | 1 | 2 | 2 |  | 2 | 0 |  | 0 | 2 | 19 (45%) |
| Labbe et al. (2002) | 3 | 3 | 2 | 0 | 2 | 2 | 3 | 3 | 1 | 2 |  | 3 | 3 |  | 0 | 2 | 29 (69%) |
| Cozart et al. (2017) | 2 | 3 | 3 | 0 | 2 | 2 | 2 | 2 | 1 | 3 |  | 3 | 1 |  | 0 | 2 | 26 (62%) |
| Kanters et al. (2013) | 3 | 2 | 3 | 0 | 2 | 2 | 2 | 3 | 1 | 2 |  | 2 | 1 |  | 0 | 2 | 25 (60%) |
| Adam et al. (2019) | 0 | 3 | 2 | 0 | 3 | 3 | 3 | 2 | 0 | 3 |  | 3 | 2 |  | 0 | 2 | 26 (62%) |
| Total score for each criterion for all papers (scored out of 69) | 32/69  46% | 59/69  86% | 59/69  86% | 0/69  0% | 43/69  62% | 47/69  68% | 37/69  54% | 43/69  62% | 19/69  28% | 52/69  75% |  | 47/69  68% | 19/69  28% |  | 5/69  7% | 37/69  54% | 0 High  13 Medium  10 Low |
| **Mixed methods studies** | Framework | Aims | Setting | Sample size | Target sample | Procedure | Tools | Recruitment | Validity of tools | Data Collection Fit (Quant) | Data Collection Fit (Qual) | Analysis fit | Analytical method | Analytical process (Qual) | User involvement | Strengths/ limitations | Score out of 48 (%) |
| Shapiro et al. (2019) | 0 | 3 | 1 | 0 | 1 | 2 | 1 | 2 | 0 | 2 | 1 | 1 | 1 | 1 | 2 | 1 | 19 (40%) |
| Schadewald, Kimball & Ou (2017) | 1 | 2 | 1 | 0 | 1 | 2 | 3 | 1 | 2 | 2 | 2 | 2 | 1 | 0 | 0 | 1 | 21 (44%) |
| Needham et al. (2013) | 1 | 3 | 2 | 3 | 2 | 2 |  | 2 | 1 | 3 | 1 | 3 | 2 | 0 | 0 | 2 | 30 (63%) |
| Malcolm et al. (2012) | 1 | 3 | 3 | 0 | 2 | 3 | 2 | 2 | 2 | 2 | 3 | 2 | 3 | 0 | 3 | 2 | 33 (79%) |
| Spilkin &Ballantyne (2007) | 3 | 3 | 1 | 0 | 2 | 2 | 3 | 2 | 3 | 2 | 2 | 1 | 0 | 1 | 1 | 3 | 29 (69%) |
| Total score for each criterion for all papers (out of 15) | 6/15  40% | 14/15  93% | 8/15  53% | 3/15  20% | 8/15  53% | 11/15  73% | 12/15  80% | 9/15  60% | 8/15  53% | 11/15  73% | 9/15  60% | 9/15  60% | 7/15  47% | 2/15  13% | 6/15  40% | 9/15  60% | 0 High  3 Medium  2 Low |
| **Qualitative studies** | Framework | Aims | Setting | Sample size | Target sample | Procedure | Tools | Recruitment | Validity of tools (Quant) | Data Collection Fit (Quant) | Data Collection Fit | Analysis fit | Analytical method | Analytical process | User involvement | Strengths/ limitations | Score out of 42 (%) |
| Porter et al. (2020) | 2 | 3 | 3 | 1 | 2 | 3 | 1 | 2 |  |  | 2 | 3 | 1 | 2 | 3 | 2 | 30 (71%) |
| Zengin, Yayan & Akinci (2020) | 2 | 3 | 2 | 1 | 2 | 3 | 3 | 3 |  |  | 3 | 3 | 3 | 2 | 2 | 2 | 34 (81%) |
| Harrington et al. (2019) | 3 | 2 | 3 | 0 | 1 | 3 | 3 | 2 |  |  | 3 | 3 | 1 | 0 | 3 | 2 | 29 (69%) |
| Somanadhan & Larkin (2016) | 3 | 3 | 3 | 0 | 2 | 2 | 3 | 2 |  |  | 0 | 3 | 3 | 0 | 0 | 2 | 26 (62%) |
| Soni-Jaiswal et al. (2016) | 2 | 3 | 2 | 0 | 2 | 2 | 3 | 2 |  |  | 3 | 3 | 3 | 0 | 0 | 2 | 28 (67%) |
| de Ru, Bouwman, Wijburg & van Zweiten (2012) | 2 | 2 | 3 | 0 | 2 | 2 | 2 | 3 |  |  | 3 | 0 | 1 | 3 | 3 | 1 | 28 (67%) |
| Young &Harper (1981) | 0 | 2 | 3 | 0 | 2 | 1 | 1 | 2 |  |  | 2 | 0 | 0 | 0 | 0 | 1 | 14 (33%) |
| Pruniski, Lisi & Ali (2018) | 2 | 2 | 2 | 0 | 2 | 2 | 0 | 2 |  |  | 2 | 2 | 0 | 3 | 0 | 2 | 21 (50%) |
| Henderson, Packman & Packman (2009) | 1 | 3 | 2 | 0 | 2 | 2 | 3 | 2 |  |  | 3 | 2 | 1 | 1 | 3 | 2 | 27 (64% |
| Freedman (2013) | 3 | 3 | 3 | 0 | 2 | 2 | 3 | 2 |  |  | 3 | 3 | 0 | 0 | 0 | 3 | 27 (64%) |
| Total score for each criterion for all papers (scored out of 30) | 20/30  67% | 26/30  87% | 26/30  87% | 2/30  7% | 19/30  63% | 24/30  80% | 22/30  73% | 22/30  73% |  |  | 24/30  80% | 22/30  80% | 13/30  43% | 11/30  37% | 15/30  50% | 19/30  63% | 1 High  8 Medium  1 Low |

*Key: Individual rating: 0 = Not at all (not present), 1 = Slightly (Briefly reported), 2 = Moderately (reported, but incomplete), 3 = Complete (reported in full). Overall quality rating: high quality, moderate quality, low qualit*
